# Identification of an Immune-Related Nine-lncRNA Signature Predictive of Overall Survival in Colon Cancer

**DOI:** 10.3389/fgene.2020.00318

**Published:** 2020-04-30

**Authors:** Yilin Lin, Xiaoxian Pan, Zhihua Chen, Suyong Lin, Shaoqin Chen

**Affiliations:** ^1^Gastrointestinal Surgery, The First Affiliated Hospital of Fujian Medical University, Fuzhou, China; ^2^Department of Radiotherapy, The First Affiliated Hospital of Fujian Medical University, Fuzhou, China

**Keywords:** colon cancer, immune, LncRNA, signature, prognosis

## Abstract

Growing evidence suggests that immune-related genes (IRGs) and long non-coding RNAs (lncRNAs) can serve as prognostic markers of overall survival (OS) in patients with colon cancer. This study aimed to identify an immune-related lncRNA signature for the prospective assessment of prognosis in these patients. Gene expression and clinical data of colon cancer patients were downloaded from The Cancer Genome Atlas (TCGA). Immune-related lncRNAs were identified by a correlation analysis between IRGs and lncRNAs. In total, 447 samples were divided into a training cohort (224 samples) and a testing cohort (223 samples). Univariate, lasso and multivariate Cox regression analyses identified an immune-related nine-lncRNA signature closely related to OS in colon cancer patients in the training dataset. A risk score formula involving nine immune-related lncRNAs was developed to evaluate the prognostic value of the lncRNA signature in the training dataset. Colon cancer patients with a high risk score had poorer OS than those with a low risk score. A multivariate Cox regression analysis confirmed that the immune-related nine-lncRNA signature could be an independent prognostic factor in colon cancer patients. The results were further confirmed in the testing cohort and the entire TCGA cohort. Furthermore, a gene set enrichment analysis revealed several pathways with significant enrichment in the high- and low-risk groups that may be helpful in formulating clinical strategies and understanding the underlying mechanisms. Finally, a quantitative real-time polymerase chain reaction assay found that the nine lncRNAs were significantly differentially expressed in colon cancer cell lines. The results of this study indicate that this signature has important clinical implications for improving predictive outcomes and guiding individualized treatment in colon cancer patients. These lncRNAs could be potential biomarkers affecting the prognosis of colon cancer.

## Introduction

Colon cancer is a common malignant tumor of the digestive tract. A study found that the incidence and mortality of colon cancer have gradually increased in recent years (Bray et al., 2018). The current diagnostic strategies for colon cancer mainly include biopsy, laboratory examination, and colonoscopy (Cross et al., 2019; Kawamura et al., 2019). Therefore, establishing a reliable risk assessment model is urgently needed to assess the survival prognosis of patients with colon cancer and optimize clinical treatment decisions. In recent years, genomic research has greatly enhanced our understanding of the mechanism underlying colon cancer development (Coebergh van den Braak et al., 2018) and revealed the potential value of molecular biomarkers in the diagnosis and evaluation of survival prognosis in patients with colon cancer (Galamb et al., 2019; Xu et al., 2019). Therefore, individualized treatment plans can be tailored to different patients with colon cancer to improve their survival prognosis.

Long non-coding RNAs (lncRNAs) constitute a class of non-coding RNAs that are more than 200 nucleotides in length and play an important role in the development of cancer (Geisler and Coller, 2013). Studies have shown that the expression or dysfunction of lncRNAs is closely related to the occurrence of human diseases, including cancers (Bermudez et al., 2019; Chi et al., 2019). A previous study found that the lncRNA HOTAIR promotes colon cancer development by downregulating miRNA-34a (Peng C. L. et al., 2019). The lncRNA FENDRR was found to inhibit colon cancer development by inhibiting the SOX4 protein (Liu and Du, 2019). In addition, a study found that the lncRNA CYTOR promotes metastasis in colon cancer through Wnt/β-catenin signaling (Yue et al., 2018). These studies indicate that the expression levels of lncRNAs affect colon cancer development and survival prognosis.

In recent decades, studies have shown that the immune system plays a key role in the development of tumors (Marshall and Djamgoz, 2018; Popovic et al., 2018). The expression and dysregulation of immune-related genes (IRGs) are involved in the regulation of the immune system. The activation of the JAK/STAT3 signaling pathway restores natural killer cell function and improves immune defense after cerebral ischemia (Jin et al., 2018). A related study found that galectin-1 restores immune tolerance to liver transplant patients by activating hepatic stellate cells (Jiang et al., 2018). Interferon regulatory factor 8 binds the Spp1 promoter to inhibit osteopontin expression in colonic epithelial cells, thereby increasing survival in colon cancer patients (Klement et al., 2018). These studies show that IRGs can be used to characterize the infiltration of immune cells in tumors. Therefore, advanced immunotherapy is expected to provide an alternative treatment for colon cancer patients.

In this study, we identified the expression of immune-related lncRNAs in 447 patients with colon adenocarcinoma. Cox regression models and lasso regression models were used to identify an immune-related lncRNA signature that was used to construct prognostic models. We identified an immune-related nine-lncRNA signature associated with the prognosis of colon cancer patients in the training cohort that performed well in the testing cohort and the entire The Cancer Genome Atlas (TCGA) cohort. Finally, we further verified that these nine lncRNAs were significantly differentially expressed between colon cancer cell lines and colon cell lines by a quantitative real-time polymerase chain reaction (qRT-PCR) test. This immune-related nine-lncRNA signature not only can improve the ability to predict prognosis in patients with colon cancer but also can promote better clinical strategies and elucidate the underlying mechanisms.

## Materials and Methods

### Data Acquisition and Processing

The RNA sequencing data and clinical data of colon adenocarcinoma samples were downloaded from TCGA^1^ based on the Illumina (San Diego, CA, United States) HiSeq 2000 RNA sequencing platform. The workflow type is counts. This dataset contains 488 colon adenocarcinoma tissues and 42 adjacent normal tissues. Samples with incomplete survival data were removed, and finally, 447 samples with complete clinical information were included in the subsequent analysis. The Ensemble IDs of the lncRNAs in TCGA database were extracted from the GENCODE project^2^ (Frankish et al., 2019). The R package (Auckland City, New Zealand) “limma” and “voom” function were used to normalize the data (Ritchie et al., 2015). The IRGs were obtained from the Molecular Signatures Database v4.0 (Immune system process M13664, Immune response M19817)^3^ (Barbie et al., 2009; Wei et al., 2019).

### Identification of Immune-Related lncRNAs

The immune-related lncRNAs were identified by a Pearson correlation analysis between the IRGs and lncRNA expression levels in the colon cancer samples (Pearson correlation coefficient >0.4, *p* < 0.001). The R package “caret” was used to randomly divide the samples into the training cohort (224 samples) and the testing cohort (223 samples) (Kuhn, 2019). The expression levels of the immune-related lncRNAs were obtained in the training cohort, testing cohort, and entire TCGA cohort.

### Construction of an Immune-Related lncRNA Signature Associated With Prognosis

The R packages “survival,” “survminer,” and “forestplot” were used to perform a univariate Cox regression analysis and multivariate Cox regression analysis (Alboukadel and Kosinski, 2019; Max and Lumley, 2019). The R package “glmnet” was used to perform a lasso regression analysis (Jerome et al., 2010). Immune-related lncRNAs with a significant prognostic value (*p* < 0.01) were screened. In the multivariate Cox regression analysis, the Akaike information criteria had a minimum value of 344.96 as the best cutoff point. The immune-related lncRNA signature is expressed as follows: risk score = (coefficient _*lncRNA*__1_ × lncRNA1 expression) + (coefficient _*lncRNA*__2_ × expression of lncRNA2) + … + (coefficient _*lncRNAn*_ × expression lncRNAn). The median risk score served as a cutoff value to classify the patients in the training cohort into the high- and low-risk groups. The testing cohort was also divided into high- and low-risk groups using the same cutoff value. The R package “survival” was used to plot Kaplan–Meier survival curves. The R package “survivalROC” was used to investigate the prognostic value of the immune-related lncRNA signature over time (Patrick and Saha-Chaudhuri, 2013). A two-sided log-rank *p* < 0.05 was considered significant in the survival analysis.

### Independence of Prognostic Factors From Other Clinical Parameters in TCGA

The complete information of the 447 patients included relevant clinical data for the univariate and multivariate Cox regression analyses. *p* < 0.05 was considered statistically significant.

### Construction of a Predictive Nomogram

A prognostic model was constructed by obtaining the independent prognostic factors, and a time-dependent receiver operating characteristic (ROC) curve was used to compare the models. The most appropriate independent prognostic factors were chosen for the prognostic model to construct a nomogram in the entire TCGA cohort. The calibration plot and concordance index (C-index) were used to investigate the calibration and the discrimination of the nomogram (by a bootstrap method with 1,000 resamples) (Iasonos et al., 2008).

### Gene Set Enrichment Analysis

To understand the Kyoto Encyclopedia of Genes and Genomes (KEGG) pathways of the immune-related lncRNA signature, a gene set enrichment analysis (GSEA) was used to analyze the enrichment terms in the entire TCGA cohort. GSEA software version 4.1.1 (Cambridge, MA, United States) was used to perform the GSEA, and *p* < 0.05 was considered statistically significant.

### Gene Expression Profiling Interactive Analysis Dataset

Gene Expression Profiling Interactive Analysis^4^ is a cancer data website. The content that can be analyzed also covers many aspects, including single genes, multiple genes, cancer types, and so on (Tang et al., 2017). Using this database, the expression of each lncRNA in normal tissues and cancer tissues can be obtained.

### Comparison of the Immune-Related lncRNA Signature With Other Colon Cancer Prognostic Models

To determine whether this immune-related lncRNA signature is better than other models, we compared (Zhou et al., 2018) six-lncRNA signature, (Xue et al., 2017) two-lncRNA signature, (Zhang et al., 2019) five-lncRNA signature, and (Zeng et al., 2017) four-lncRNA signature. We obtained the lncRNAs in these models from the literature and constructed 1-, 3-, and 5-year ROC curves and survival curves for the entire TCGA cohort. These lncRNA signatures were compared with the immune-related lncRNA signature identified in this study to evaluate their pros and cons.

### Cell Culture

A colon cell line (CCD-841CoN) and colon cancer cell lines (HCT116 and SW480) were purchased from the Cell Bank of the Chinese Academy of Sciences (Shanghai, China). The CCD-841CoN cells were grown in High Glucose Dulbecco’s Modified Eagle Medium, the HCT116 cells were grown in McCoy’s 5A medium, and the SW480 cells were grown in RPMI-1640 medium (Gibco, Carlsbad, CA, United States) containing 10% fetal bovine serum (Gibco). The cells were grown at 37°C with 5% CO_2_.

### Detection of lncRNA Expression in Cell Lines

The RNA was extracted with TRIzol reagent (TransGen Biotech, Beijing, China), and then, the expression levels of nine lncRNAs were detected by qRT-PCR. The qRT-PCR assay was performed as follows: RNA was detected using a reverse transcription kit (TaKaRa, Dalian, China) and an amplification kit (TaKaRa) following the manufacturer’s instructions. GAPDH was used as an internal control. The reaction mixture contained 10 μL of SYBR Premix Ex Taq 2, 1 μL of each primer, 2 μL of the cDNA template, and 6 μL of ddH_2_O for a final volume of 20 μL. The thermal cycling parameters used for amplification were as follows: a denaturation step at 95°C for 30 s, followed by 40 cycles at 95°C for 5 s, and a final holding step at 60°C for 34 s. The relative gene expression levels were evaluated with Data Assist software version 3.0 (Applied Biosystems, Foster City, CA, United States). The relative expression levels were determined according to the 2^–ΔΔ*CT*^ method. The assays were performed in triplicate. The primer sequences for each gene are shown in Supplementary Table S1.

### Statistical Analysis

The statistical analyses were performed using SPSS software version 17.0 (Armonk, New York, United States) and R software version 3.6.0 (Auckland City, New Zealand). Pearson χ^2^ test or Fisher exact test were used to explore the qualitative variables as appropriate.

## Results

### Identification of Immune-Related lncRNAs

In total, 761 immune-related lncRNAs were identified in the 447 colon adenocarcinoma samples. The R package “caret” was used to randomly divide the samples into the training cohort and the testing cohort. The clinical features of the colon cancer patients are shown in Table 1. The expression profiles of the 761 immune-related lncRNAs in the training cohort were used to construct a prognostic model.

**TABLE 1:** Clinical features of the colon cancer patients in each cohort.

**Variables**	**Training cohort (*n* = 224)**	**Testing cohort (*n* = 223)**	***P***	**Entire TCGA cohort (*n* = 447)**
**Age (years)**				
≤65	92	107	0.1539	199
≥65	132	116		248
**Survival status**				
Living	180	189	0.2621	369
Dead	44	34		78
**Gender**				
Female	104	98	0.6350	202
Male	120	125		245
**Tumor invasion (T)**				
T1	4	10	0.4271	14
T2	40	41		81
T3	157	148		305
T4	23	23		46
Unknown	0	1		1
**Lymph node (N)**				
N0	130	131	0.2608	261
N1	49	60		109
N2	44	32		76
Unknown	1	0		1
**Metastasis (M)**				
M0	172	163	0.3982	335
M1	28	38		66
Unknown	24	22		46
**Tumor stage**				
Stage I	36	44	0.3353	80
Stage II	84	81		165
Stage III	65	55		120
Stage IV	29	38		67
Unknown	10	5		15

### Construction and Validation of the Immune-Related lncRNA Signature in TCGA

**FIGURE 1:**
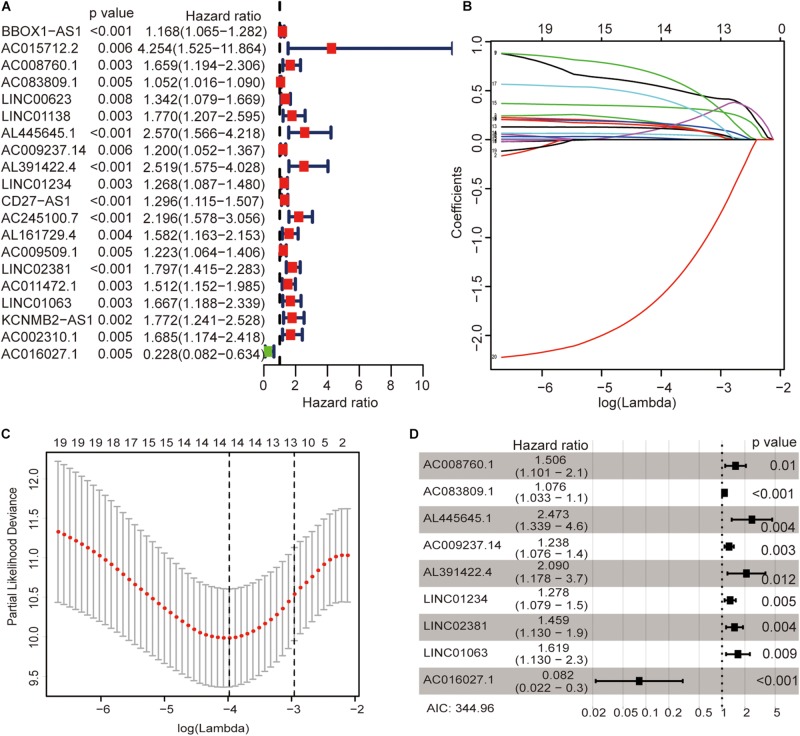
Identification of immune-related lncRNAs associated with colon cancer prognosis. Univariate Cox regression models identified 20 immune-related lncRNAs associated with OS **(A)**. Fourteen immune-related lncRNAs were identified by a lasso regression analysis **(B,C)**. Nine immune-related lncRNAs were identified for the construction of a prognostic model by a multivariate Cox regression analysis **(D)**.

**FIGURE 2:**
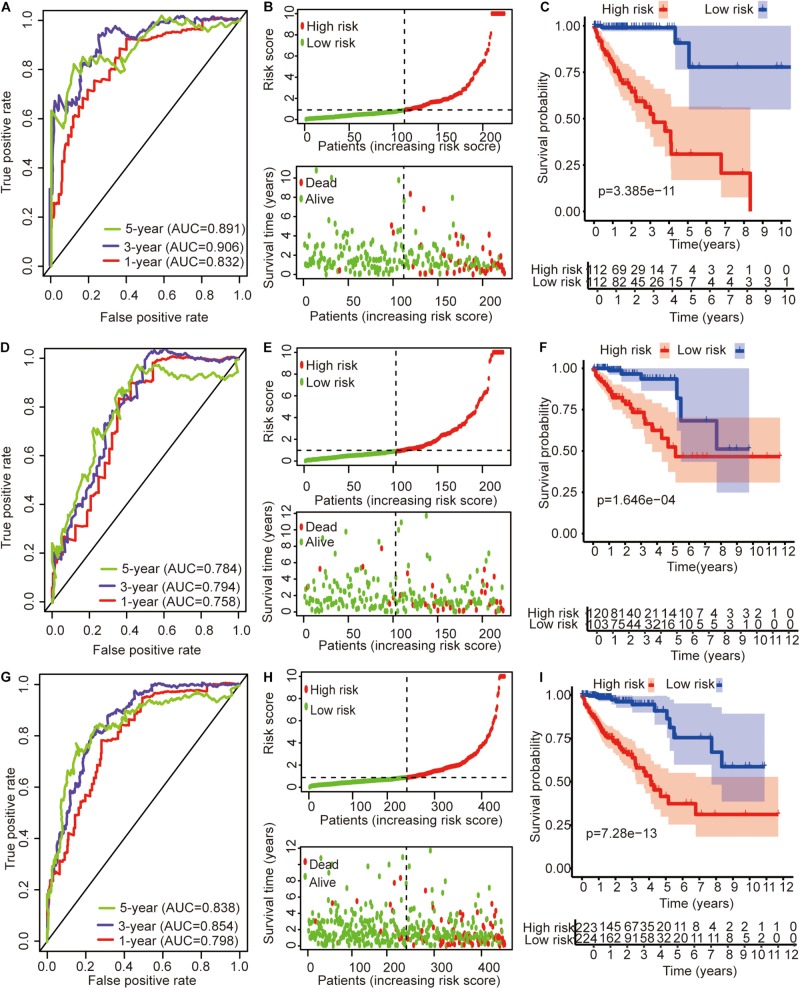
Time–ROC curve analysis, risk score analysis, and Kaplan–Meier curve survival analysis of the immune-related lncRNA signature in colon cancer. **(A)** Time–ROC curve analysis, **(B)** risk score, and **(C)** Kaplan–Meier survival curve of the immune-related lncRNA signature in the training cohort. **(D)** Time–ROC curve analysis, **(E)** risk score, and **(F)** Kaplan–Meier survival curve of the immune-related lncRNA signature in the testing cohort. **(G)** Time–ROC curve analysis, **(H)** risk score, and **(I)** Kaplan–Meier survival curve of the immune-related lncRNA signature in the entire TCGA cohort.

The univariate Cox regression models identified 20 immune-related lncRNAs associated with overall survival (OS) (Figure 1A). Fourteen immune-related lncRNAs were identified by a lasso regression analysis (Figures 1B,C). Nine immune-related lncRNAs were identified for the construction of the prognostic model by a multivariate Cox regression analysis (Figure 1D). The results of the univariate and multivariate Cox regression analyses of the nine immune-related lncRNAs in the training cohort are shown in Table 2. The nine immune-related lncRNAs include AC008760.1, AC083809.1, AL445645.1, AC009237.14, AL391422.4, LINC01234, LINC02381, LINC01063, and AC016027.1. The model is expressed as follows: risk score = 0.409 × expression of AC008760.1 + 0.074 × expression of AC083809.1 + 0.906 × expression of AL445645.1 + 0.214 × expression of AC009237.14 + 0.737 × expression of AL391422.4 + 0.245 × expression of LINC01234 + 0.378 × expression of LINC02381 + 0.482 × expression of LINC01063-2.506 × expression of AC016027.1. The patients were divided into high- and low-risk groups by the median risk score (0.909), which served as a cutoff value. The model constructed with the nine-lncRNA immune signature showed that the area under the curve (AUC) values of the time–ROC curve of the 1-, 3-, and 5-year OS were 0.832, 0.906, and 0.891, respectively (Figure 2A). As the risk score increased, the patient mortality rate gradually increased (Figure 2B). The OS in the high-risk group was significantly worse than that in the low-risk group (Figure 2C, *p* < 0.0001). Then, the prognostic model was validated in the testing cohort, and this model showed that the AUC values of the 1-, 3-, and 5-year OS were 0.758, 0.794, and 0.784, respectively (Figure 2D). As the risk score increased, the patient mortality rate gradually increased (Figure 2E). According to the median risk score obtained from the training cohort (0.909), the patients in the testing cohort were also divided into high- and low-risk groups. The OS in the high-risk group was significantly worse than that in the low-risk group (Figure 2F, *p* < 0.001). Similarly, in the entire TCGA cohort, the results showed that the AUC values of the 1-, 3-, and 5-year OS were 0.758, 0.854, and 0.838, respectively (Figure 2G). As the risk score increased, the patient mortality rate gradually increased (Figure 2H). The OS in the high-risk group was significantly worse than that in the low-risk group (Figure 2I, *p* < 0.0001). These results indicate that the prognostic model is moderately sensitive and specific.

**TABLE 2:** Univariate and multivariate Cox regression analyses of nine immune-related lncRNAs in the training cohort.

**Variables**	**Ensemble ID**	**Univariate analysis**		**Multivariate analysis**		
		**HR 95% CI**	***P***	**Coefficient**	**HR (95% CI)**	***P***
AC008760.1	ENSG00000269680	1.659 (1.194–2.304)	0.003	0.409	1.506 (1.101–2.059)	0.01
AC083809.1	ENSG00000277247	1.052 (1.016–1.090)	0.005	0.074	1.076 (1.033–1.122)	< 0.001
AL445645.1	ENSG00000224992	2.570 (1.566–4.218)	< 0.001	0.906	2.473 (1.339–4.569)	0.004
AC009237.14	ENSG00000272913	1.200 (1.052–1.367)	0.006	0.214	1.238 (1.076–1.425)	0.003
AL391422.4	ENSG00000270504	2.519 (1.575–4.028)	< 0.001	0.737	2.090 (1.178–3.711)	0.012
LINC01234	ENSG00000249550	1.268 (1.087–1.480)	0.002	0.245	1.278 (1.079–1.513)	0.005
LINC02381	ENSG00000250742	1.797 (1.415–2.283)	< 0.001	0.378	1.459 (1.130–1.884)	0.004
LINC01063	ENSG00000232065	1.667 (1.188–2.339)	0.003	0.482	1.619 (1.130–2.320)	0.009
AC016027.1	ENSG00000225335	0.228 (0.082–0.634)	0.005	–2.506	0.082 (0.022–0.305)	< 0.001

### Correlation Between the Clinicopathologic Characteristics and the Immune-Related lncRNA Signature in Colon Cancer

**FIGURE 3:**
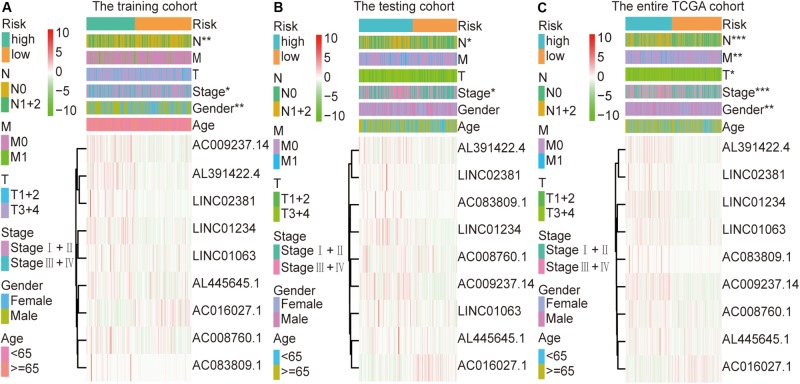
Relationship between the risk score and clinical characteristics of colon cancer in three cohorts. **(A)** Risk scores were significantly associated with gender, lymph nodes, and clinical stage in the training cohort. **(B)** Risk scores were significantly associated with lymph nodes and clinical stage in the testing cohort. **(C)** Risk scores were significantly associated with gender, lymph nodes, metastases, tumor depth, and clinical stage in the entire TCGA cohort. *p < 0.05, **p < 0.01, ***p < 0.001.

The colon cancer patients were divided into two groups (high- and low-risk groups) according to the risk score of the model in the training cohort, testing cohort, and entire TCGA cohort (Figure 3 and Table 3). The colon cancer patient information included age, gender, tumor stage, tumor invasion, lymph node, and distant metastasis. The risk scores were found to be significantly correlated with gender, the tumor stage, and lymphatic metastasis in the training cohort. In the testing cohort, the risk scores were significantly correlated with the tumor stage and lymphatic metastasis. Similarly, the risk scores were significantly associated with gender, the tumor stage, tumor invasion, lymphatic metastasis, and distant metastasis in the entire TCGA cohort. Combining the results of the above three cohorts showed a significant association between the immune-related lncRNA signature model and the tumor stage and lymphatic metastasis.

**TABLE 3:** Correlation between clinicopathologic characteristics and the immune-related lncRNA signature in colon cancer.

**Variables**	**Training cohort (*n* = 224)**			**Testing cohort (*n* = 223)**			**Entire TCGA (*n* = 447)**		
			
	**High risk**	**Low risk**	***P***	**High risk**	**Low risk**	***P***	**High risk**	**Low risk**	***P***
**Age (years)**									
≤65	42	50	0.277	52	55	0.134	92	107	0.166
>65	70	62		68	48		131	117	
**Gender**									
Female	42	62	0.007	52	46	0.842	87	115	0.009
Male	70	50		68	57		136	109	
**Tumor stage**									
Stage I + II	51	69	0.03	58	67	0.020	99	146	<0.0001
Stage III + IV	54	40	58	35	114	73			
Unknown	7	3		4	1		10	5	
**Tumor invasion (T)**									
T 1 + 2	18	26	0.179	22	29	0.075	38	57	0.032
T 3 + 4	94	86		98	73		184	167	
Unknown	0	0		0	1		1	0	
**Lymph node (N)**									
N0	54	76	**0.004**	62	69	0.021	105	156	<0.0001
N 1 + 2	57	36		58	34		117	68	
Unknown	1	0		0	0		1	0	
**Metastasis (M)**									
M0	77	95	0.055	86	77	0.246	152	183	0.007
M1	18	10		24	14		42	24	
Unknown	17	7		10	12		29	17	

### Identification of Independent Prognostic Factors

In the training cohort, the univariate and multivariate Cox regression analyses showed that the tumor stage and risk score were independent prognostic factors for OS in colon cancer patients (Table 4). In the testing cohort, the univariate and multivariate Cox regression analyses showed that age, the tumor stage, and the risk score were independent prognostic factors for OS (Table 4). Similarly, in the entire TCGA cohort, age, the tumor stage, and the risk score were also independent prognostic factors for OS (Table 4). Based on the above results, the *p* values of only the tumor stage and risk score were less than 0.05 in the three cohorts. This result suggests that the tumor stage and risk score may be independent prognostic factors for OS in colon cancer patients.

**TABLE 4:** Univariate and multivariate Cox regression analyses of the clinical features in each cohort.

**Variables**	**Univariate analysis**		**Multivariate analysis**	
	**HR 95% CI**	***P***	**HR (95% CI)**	***P***
**Training cohort**				
Age	1.025 (0.997–1.054)	0.079	1.037 (1.007–1.067)	0.014
Gender	1.444 (0.786–2.654)	0.237	1.102 (0.590–2.058)	0.760
Stage	1.973 (1.504–2.589)	< 0.001	1.852 (1.413–2.428)	< 0.001
Risk score	1.020 (1.013–1.028)	< 0.001	1.021 (1.013–1.029)	< 0.001
**Testing cohort**				
Age	1.038 (1.005–1.073)	0.025	1.053 (1.016–1.092)	0.005
Gender	0.935 (0.472–1.850)	0.846	0.668 (0.326–1.368)	0.270
Stage	2.061 (1.491–2.849)	< 0.001	2.158 (1.554–2.998)	< 0.001
Risk score	1.019 (1.010–1.028)	< 0.001	1.021 (1.011–1.030)	< 0.001
**Entire TCGA cohort**				
Age	1.032 (1.010–1.054)	0.004	1.033 (1.011–1.056)	0.003
Gender	1.179 (0.750–1.854)	0.477	0.992 (0.624–1.576)	0.973
Stage	1.982 (1.612–2.436)	< 0.001	1.909 (1.553–2.348)	< 0.001
Risk score	1.053 (1.040–1.067)	< 0.001	1.045 (1.031–1.059)	< 0.001

### Construction of the Prognostic Nomogram

**FIGURE 4:**
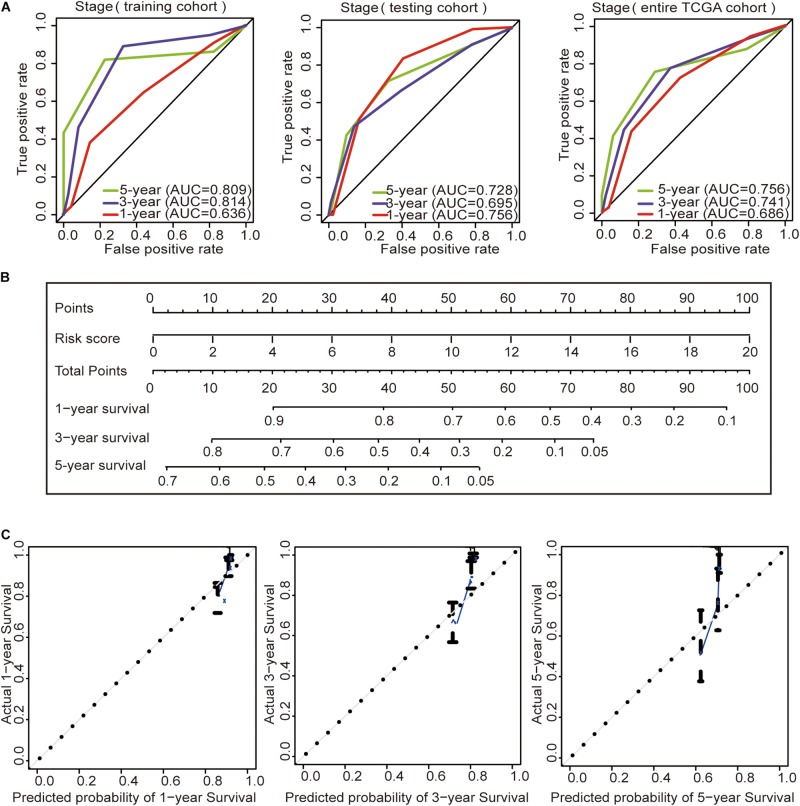
Construction of the prognostic model and GSEA. **(A)** Time–ROC curves of the tumor stage prognostic model were compared in terms of the 1-, 3-, and 5-year OS in each cohort. **(B)** Construction of the nomogram was based on the risk score in the entire TCGA cohort. **(C)** Calibration plot for the internal validation of the nomogram.

Subsequently, we constructed a prognostic model including the tumor stage, and the AUC values of the time–ROC curve were used to assess the accuracy of the model (Figure 4A). In the training cohort, the AUC values of the 1-, 3-, and 5-year OS were 0.636, 0.814, and 0.809, respectively. In the testing cohort, the AUC values of the 1-, 3-, and 5-year OS were 0.756, 0.695, and 0.728, respectively. In the entire TCGA cohort, the AUC values of the 1-, 3-, and 5-year OS were 0.686, 0.741, and 0.756, respectively. This result indicates that the AUC values of the tumor staging model are lower than those of the prognostic model constructed with the immune-related lncRNA signature of 1-, 3-, and 5-year OS. Prognostic models constructed with the immune-related lncRNA signature may be better predictors of OS in colon cancer patients. Therefore, we constructed a nomogram to predict OS in patients with colon cancer based on the risk scores (Figure 4B). The calibration plots showed that the performance of the nomogram was the best in predicting the 1-, 3-, and 5-year OS (Figure 4C).

### Gene Set Enrichment Analysis

**FIGURE 5:**
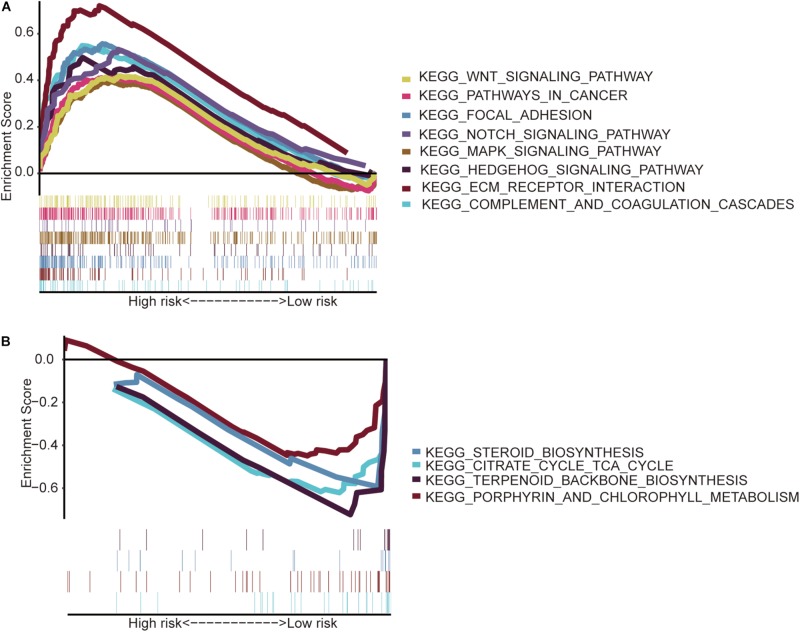
Gene set enrichment analysis. **(A)** High-risk patients with eight significantly enriched KEGG pathways in the entire TCGA cohort by GSEA. **(B)** Four significantly enriched KEGG pathways in the low-risk patients were analyzed by GSEA in the entire TCGA cohort.

The GSEA indicated that the immune-related lncRNA signature was mainly enriched in the complement and coagulation cascades, extracellular matrix (ECM) receptor interaction, focal adhesion, hedgehog signaling pathway, mitogen-activated protein kinase (MAPK) signaling pathway, notch signaling pathway, pathways in cancer and Wnt signaling pathway in the high-risk patients (Figure 5A) and the main citrate cycle, tricarboxylic acid cycle, porphyrin and chlorophyll metabolism, steroid biosynthesis, and terpenoid backbone biosynthesis in the low-risk patients (Figure 5B).

### Comparison of the Immune-Related lncRNA Signature With Other Colon Cancer Prognostic Models

**FIGURE 6:**
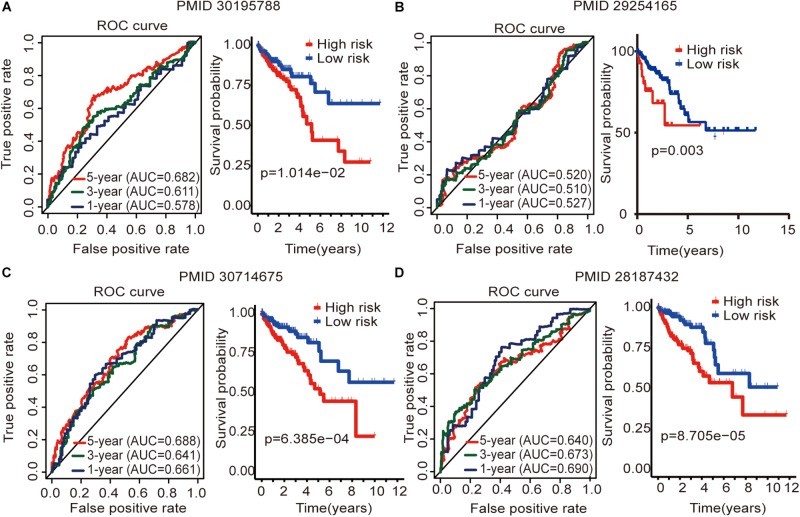
Four lncRNA signatures predict prognosis of colon cancer in the entire TCGA cohort. **(A)** Zhou et al. (2018) (PMID30195788) six-lncRNA signature in the entire TCGA cohort. **(B)** Xue et al. (2017) (PMID29254165) two-lncRNA signature in the entire TCGA cohort. **(C)** Zhang et al. (2019) (PMID30714675) five-lncRNA signature in the entire TCGA cohort. **(D)** Zeng et al. (2017) (PMID28187434) four-lncRNA signature in the entire TCGA cohort. The AUC value of the ROC curve is used to evaluate the ability to predict, and the survival curve is used to analyze the difference in prognosis between the high and low expression groups.

To determine whether this immune-related lncRNA signature is better than other models, we compared (Zhou et al., 2018) six-lncRNA signature, (Xue et al., 2017) two-lncRNA signature, (Zhang et al., 2019) five-lncRNA signature, and (Zeng et al., 2017) four-lncRNA signature. The prognosis of the high-risk group and the low-risk group significantly differed across these four models (Figure 6). Zhou et al. (2018) six-lncRNA signature shows that the AUC values of the 1-, 3-, and 5-year OS are 0.578, 0.611, and 0.682, respectively (Figure 6A). Zhang et al. (2019) five-lncRNA signature shows that the AUC values of the 1-, 3-, and 5-year OS are 0.661, 0.641, and 0.688, respectively (Figure 6C). Zeng et al. (2017)four-lncRNA signature shows that the AUC values of the 1-, 3-, and 5-year OS are 0.690, 0.673, and 0.640, respectively (Figure 6D). This result shows that all three models have a certain predictive power. However, in Xue et al. (2017) two-lncRNA signature, the AUC values of the 1-, 3-, and 5-year OS are 0.527, 0.510, and 0.520, respectively (Figure 6B). This result indicates that the model has poor predictive ability. By comparing with immune-related lncRNA signatures, we find that the accuracy of the immune-related lncRNA signature prediction is higher than that of these four models (Table 5).

**TABLE 5:** Comparison of the immune-related lncRNA signature with four other lncRNA signatures.

**Model**	**AUC**		
	**1 Year**	**3 Years**	**5 Years**
**Entire TCGA cohort**			
Immune-related lncRNA signature	0.798	0.854	0.838
Six-lncRNA signature (PMID 30195788)	0.578	0.611	0.682
Two-lncRNA signature (PMID 29254165)	0.527	0.510	0.520
Five-lncRNA signature (PMID 30714675)	0.661	0.641	0.688
Four-lncRNA signature (PMID 28187434)	0.640	0.673	0.690

### Relationship Between the Expression Levels of Nine lncRNAs and the Clinicopathological Characteristics and Survival Prognosis of Colon Cancer Patients

**FIGURE 7:**
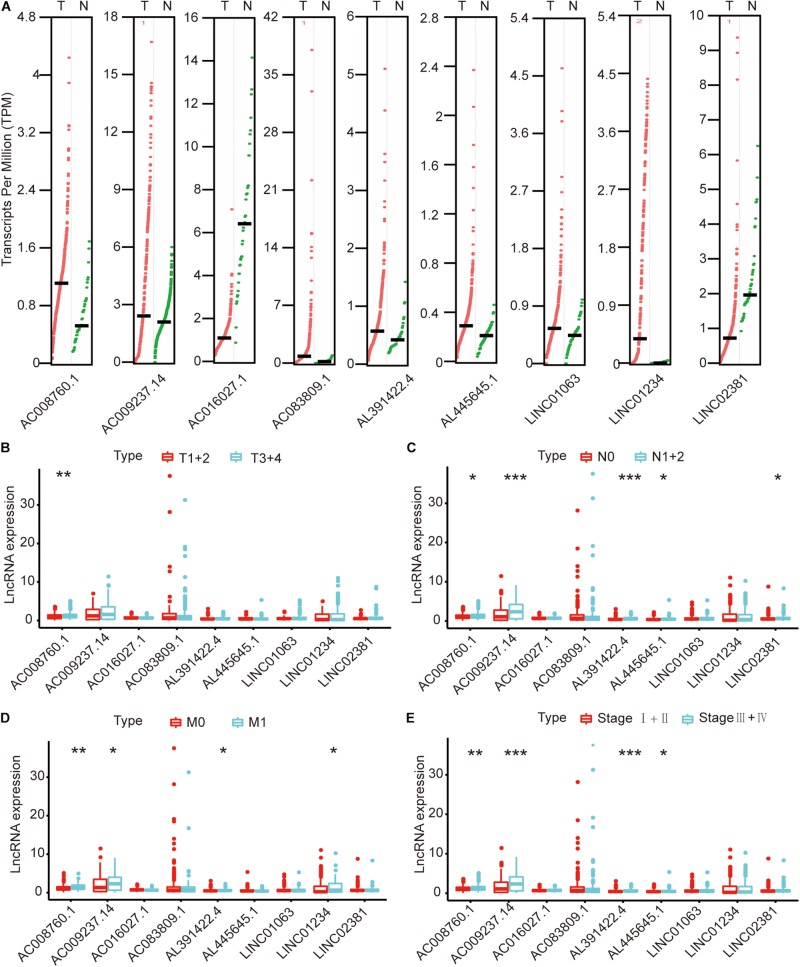
Relationship between nine lncRNAs and clinical characteristics of colon cancer in the entire TCGA cohort. **(A)** Expression levels of nine lncRNAs in the GEPIA database. **(B)** Relationship between the expression of nine lncRNAs and the depth of colon cancer tumors. **(C)** Relationship between the expression of nine lncRNAs and the number of lymphoma in colon cancer. **(D)** Relationship between the expression of nine lncRNAs and metastasis of colon cancer cells. **(E)** Relationship between the expression of nine lncRNAs and clinical stage of colon cancer. *p < 0.05, **p < 0.01, ***p < 0.001.

**FIGURE 8:**
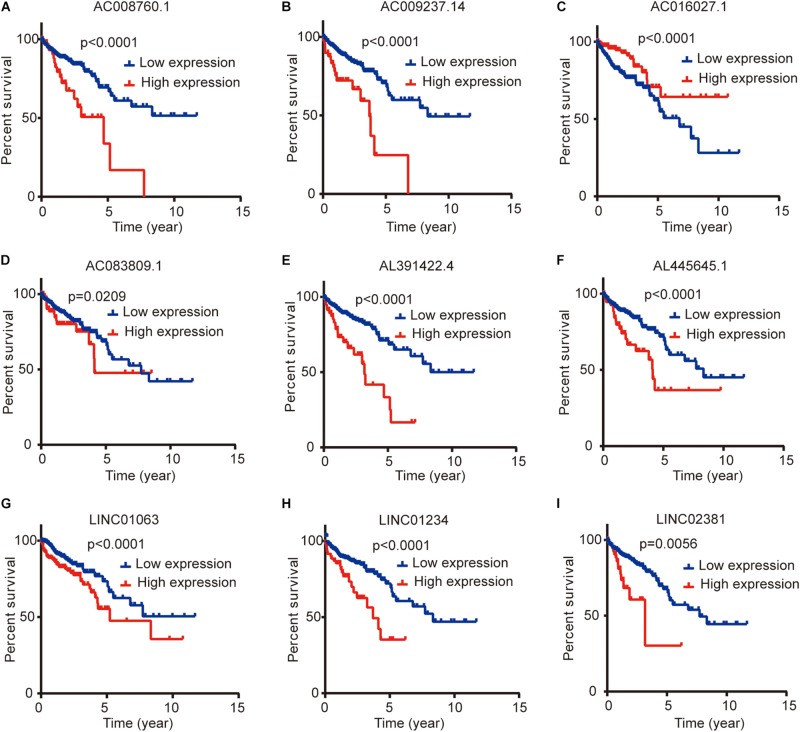
Relationship between the expression levels of nine lncRNAs and survival prognosis of colon cancer. **(A,B,D–I)** Low and high expression levels of AC008760.1, AC009237.14, AC083809.1, AL391422.4, AL445645.1, LINC01063, LINC01234, and LINC02381 are associated with significantly different prognosis, and the high expression group has a worse prognosis than the low expression group in colon cancer. **(C)** Prognosis significantly differs between groups with high expression and low expression of AC016027.1, and the low expression group has a worse prognosis than the high expression group.

We compared the lncRNA expression levels in colon cancer tissues and normal colon tissues in the GEPIA database, and the results showed that AC008760.1, AC009237.14, AC083809.1, AL391422.4, AL445645.1, LINC01063, and LINC01234 were highly expressed in colon cancer, whereas AC016027.1 and LINC02381 exhibited low expression in colon cancer (Figure 7A). The expression of AC008760.1 was significantly correlated with the depth of colon cancer invasion (Figure 7B), and the expression of AC008760.1, AL445645.1, AC009237.14, AL391422.4, and LINC02381 was significantly associated with lymph node metastasis (Figure 7C). In addition, the expression of AC008760.1, AC009237.14, AL391422.4, and LINC01234 was significantly correlated with tumor cell metastasis (Figure 7D), and the expression of AC008760.1, AL445645.1, AC009237.14, and AL391422.4 was significantly related to the clinical stage of colon cancer (Figure 7E). The survival analysis showed that a high expression of AC008760.1 (Figure 8A), AC009237.14 (Figure 8B), AC083809.1 (Figure 8D), AL391422.4 (Figure 8E), AL445645.1 (Figure 8F), LINC01063 (Figure 8G), LINC01234 (Figure 8H), and LINC02381 (Figure 8I) was associated with a poor prognosis in colon cancer. The survival analysis results show that a low expression of AC016027.1 (Figure 8C) is associated with a good prognosis.

### Expression Level of Nine lncRNAs in Cell Lines as Detected by a qRT-PCR Assay

**FIGURE 9:**
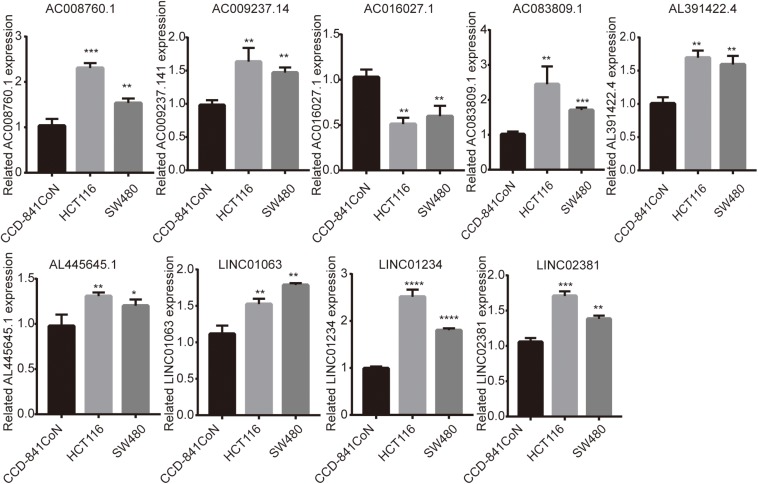
Expression of nine lncRNAs in colon cancer cell lines and colon cell lines. Expression levels of nine lncRNAs in colon cancer cell lines (HCT116 and SW480) significantly differed from those in colon cell lines (CCD-841CoN). AC008760.1, AC009237.14, AC083809.1, AL391422.4, AL445645.1, LINC01063, LINC01234, and LINC02381 were highly expressed in HCT116 and SW480. AC016027.1 was lowly expressed in HCT116 and SW480. *p < 0.05, **p < 0.01, ***p < 0.001, ****p < 0.0001.

Finally, we detected the expression levels of nine lncRNAs in CCD-841CoN, HCT116, and SW480 by a qRT-PCR assay. The results showed that AC008760.1, AC009237.14, AC083809.1, AL391422.4, AL445645.1, LINC01063, LINC01234, and LINC02381 were highly expressed in HCT116 and SW480 and lowly expressed in CCD-841CoN. There were significant differences (Figure 9). However, the expression level of AC016027.1 was low in HCT116 and SW480 and high in CCD-841CoN. There were significant differences (Figure 9).

## Discussion

Colon cancer is a common malignant tumor of the digestive tract (Bray et al., 2018). The mechanisms underlying colon cancer development and progression remain unclear. Recent studies have found that cellular immune responses are involved in the development of cancer and may be among the factors influencing poor prognosis in cancer (Li et al., 2019; Thompson et al., 2019). Studies have found that lncRNAs are important regulators of the immune response in cancer. A related study reported that tumor cells use SNHG12 to evade immune-mediated attacks and enhance the immune response (Tamang et al., 2019). The lncRNA SNHG14 is involved in the development of diffuse large B-cell lymphoma and immune escape (Zhao et al., 2019). MIR155HG is involved in immune infiltration and immune checkpoint molecular expression in various cancers and may be a biomarker of tumor prognosis (Peng L. et al., 2019). These studies indicate that immune-related lncRNAs can serve as potential markers for cancer therapy and have an important impact on the survival prognosis of cancer patients. However, biomarkers that can effectively predict the survival prognosis of colon cancer patients are still lacking. Therefore, identifying effective colon cancer biomarkers is important.

The expression profile of 761 immune-related lncRNAs in colon cancer was identified by a correlation analysis of lncRNAs and IRGs. Nine immune-related lncRNAs were associated with OS in the training cohort by univariate Cox regression, lasso regression, and multivariate Cox regression analyses. The nine immune-related lncRNAs were used to construct a signature for the prediction of OS in colon cancer patients in the training cohort (Figure 2). The risk scores generated from the expression levels of these nine immune-related lncRNAs could accurately predict the OS of the patients in the training cohort at 1 year (AUC = 0.832), 3 years (AUC = 0.906), and 5 years (AUC = 0.891) (Figure 2A). The patients were divided into high- and low-risk groups based on their risk score (Figure 2B). There was a significant difference in prognosis between the high- and low-risk groups (Figure 2C). The immune-related lncRNA signature also performed well in the testing cohort and the entire TCGA cohort (Figures 2D–I). This result further shows that the immune-related lncRNA signature we constructed has a higher accuracy than the previously reported lncRNA signature.

To further explore the relationship between this immune-related lncRNA signature and the clinical characteristics of colon cancer, we analyzed the number of samples in the high- and low-risk groups according to age, gender, the tumor stage, tumor invasion (T), lymph node (N), and metastasis (M) in the training, testing, and entire TCGA cohorts. Combining the results of the above three cohorts showed a close association between the immune-related lncRNA signature model and the tumor stage and lymphatic metastasis (Figure 3 and Table 3). By analyzing the relationship between the expression of each lncRNA and the clinical characteristics, we also found that the expression of AC008760.1, AC009237.14, and AL391422.4 is related to lymphatic metastasis in colorectal cancer, distant metastasis of cancer cells, and clinicopathological staging, with a significant correlation. This finding suggests that the expression of these three lncRNAs may be related to the development of colon cancer and may promote metastasis in tumor cells, providing a potential target for further research investigating the mechanism underlying colon cancer development and metastasis.

Then, we analyzed the relationship between age, gender, tumor stage, and risk score in patients with colon cancer by univariate Cox regression analysis and multivariate Cox analysis in the three cohorts. The results showed that both the tumor stage and risk score had *p* < 0.05 in the univariate Cox regression analysis and multivariate Cox regression analysis in the three cohorts (Table 4), suggesting that the tumor stage and risk score may be independent prognostic factors in colon cancer patients. To determine which of the two independent prognostic factors can better predict OS in colon cancer patients, we constructed a tumor stage model in the three cohorts (Figure 4A). The AUC values of the time–ROC curve were used to assess the accuracy of the model. The results show that the 1-, 3-, and 5-year prediction accuracies of this model are lower than those of the immune-related lncRNA signature in the three cohorts. Then, we constructed a nomogram with the risk score for the OS prediction of colon cancer patients in the TCGA cohort (Figure 4B). We can initially assess the 1-, 3-, and 5-year OS of each patient based on their risk score. The survival prognosis of patients can be evaluated before surgery. Therefore, patients can have a certain understanding of their condition and may be more accurately treated.

Then, the GSEA revealed several significantly enriched pathway signatures. Patients with high-risk scores are mainly enriched in the complement and coagulation cascades, ECM receptor interaction, focal adhesion, hedgehog signaling pathway, MAPK signaling pathway, notch signaling pathway, pathways in cancer, and Wnt signaling pathway (Figure 5A). Studies have reported that these pathways are closely related to the immune process (Gao et al., 2019; Hanna et al., 2019; Ramesh et al., 2019; Yang et al., 2019). Patients with low-risk scores are mainly enriched in terpenoid backbone biosynthesis, steroid biosynthesis, porphyrin and chlorophyll metabolism, and the citrate cycle (Figure 5B). From this perspective, low-risk patients may benefit more from metabolic therapy, whereas high-risk patients may benefit more from immunotherapy. However, more effort is needed to investigate this relationship. Moreover, the results shed light on the potential molecular mechanisms of signatures that provide promising directions in immunotherapy. Previous studies have developed prognostic models of lncRNA signatures for colon cancer (Xue et al., 2017; Zeng et al., 2017; Zhou et al., 2018: Zhang et al., 2019). By comparing these models, we found that the immune-related lncRNA signature based on IRGs predicts 1-, 3-, and 5-year (Figure 2) prognostic capabilities that are higher than those in previous studies of lncRNA signatures (Figure 6). It is suggested that immune-related lncRNA signatures based on IRGs can better assess the prognosis of patients with colon cancer.

Finally, we analyzed the expression level of each lncRNA. The GEPIA database showed that AC008760.1, AC009237.14, AC083809.1, AL391422.4, AL445645.1, LINC01063, and LINC01234 were highly expressed in colon cancer and that AC016027.1 has low expression in colon cancer (Figure 7A). Moreover, the survival analysis showed that AC008760.1, AC009237.14, AC083809.1, AL391422.4, AL445645.1, LINC01063, and LINC01234 overexpression is associated with a poor prognosis, whereas AC016027.1 overexpression is associated with a good prognosis (Figure 8). This finding is consistent with the results of our previous Cox regression analysis (Figure 1). In the GEPIA database, LINC02381 was lowly expressed in colon cancer (Figure 7A), but both the Cox regression analysis and survival analysis showed that LINC02381 may be a procancer factor in colon cancer (Figure 1). In addition, we finally tested the expression of LINC02381 in cell lines and found that LINC02381 was highly expressed in colon cancer cell lines (Figure 9). Therefore, we speculate that this result may be due to too few normal colon samples in the GEPIA database, and more samples may need to be collected for verification in the future. Although our results may have potentially significant clinical implications, several issues need to be noted. First, some important immune genes may have been excluded before establishing the prognostic model, which may ultimately reduce the performance of the model. In addition, although immune regulation contributes to the development of colon cancer, more complex mechanisms may affect prognosis in colon cancer and require further exploration.

In summary, the immune-related lncRNA signature showed independent prognostic significance in colon cancer. The results of this study provide a method for predicting the prognosis and survival of colon cancer patients and may provide potential lncRNA targets for immunotherapy.

## Data Availability Statement

Publicly available datasets were analyzed in this study. This data can be found here: https://cancergenome.nih.gov/, http://www.roadinstitute.org/gsea/msigdb/index.jsp.

## Ethics Statement

Ethical review and approval was not required for the study on human participants in accordance with the local legislation and institutional requirements. Written informed consent for participation was not required for this study in accordance with the national legislation and the institutional requirements.

## Author Contributions

SC sponsored the fund. YL, XP, and ZC designed research. YL and XP participated in the writing of the manuscript. SL participated in the research of data statistics and manuscript layout.

## Conflict of Interest

The authors declare that the research was conducted in the absence of any commercial or financial relationships that could be construed as a potential conflict of interest.

## Footnotes

1 https://cancergenome.nih.gov/

2 http://www.gencodegenes.org

3 http://www.broadinstitute.org/gsea/msigdb/index.jsp

4 http://gepia.cancer-pku.cn/index.html
