# Supplementary material for: Identification of an Immune-Related Nine-lncRNA Signature Predictive of Overall Survival in Colon Cancer
Source: Front Genet. 2020 Apr 30;11:318. doi: 10.3389/fgene.2020.00318 (PMC7203495; doi:10.3389/fgene.2020.00318)
Supplement: Supplementary file 1 [file Table_1.DOC]

**Table S1 Primer sequences for each gene.**

| **Gene** | **Primer sequence** | |
| --- | --- | --- |
| GAPDH | Forward | 5’-CCCTTCATTGACCTCAACTACATG-3’ |
|  | Reverse | 5’-TGGGATTTCCATTGATGACAAGC-3’ |
| AC008760.1 | Forward | 5’-CATAAACCACTGCACCCTGC-3’ |
|  | Reverse | 5’-TGGGCAGCCTGTTTTACCTT-3’ |
| AC083809.1 | Forward | 5’-AATCGATACTCGGTTGCGGT-3’ |
|  | Reverse | 5’-TGGTCAGGAGCTAATGGGGA-3’ |
| AL445645.1 | Forward | 5’-AGCTCATGACCGCCTTGAAA-3’ |
|  | Reverse | 5’-AGTCACTATGGGTGGGGTGA-3’ |
| AC009237.14 | Forward | 5’-TATTTAGGGCTGCGTTCCCC-3’ |
|  | Reverse | 5’-GACTGACCACAATGGCTGGA-3’ |
| AL391422.4 | Forward | 5’-CGGAGTGGTTTCGGGTTGTA-3’ |
|  | Reverse | 5’-TTGGAAGATGGGCAGGTGAC-3’ |
| LINC01234 | Forward | 5’-TCCCACAAAACAACCACCCA-3’ |
|  | Reverse | 5’-TGAGTCAGTGGCAGAGCAAG-3’ |
| LINC02381 | Forward | 5’-TATCACACCAAGGCCACACC-3’ |
|  | Reverse | 5’-GAATGAACAGCCGAAGCGAC-3’ |
| LINC01063 | Forward | 5’-TCACCTCCTCAAAACTGGCC-3’ |
|  | Reverse | 5’-CCTGGGCTTCGAGACAGTTT-3’ |
| AC016027.1 | Forward | 5’-ATGTTGGTCCCAAGGGCAAT-3’ |
|  | Reverse | 5’-AGCAAATTCAGGATGGGGCA-3’ |
